# Screening for phenotypic outliers identifies an unusually low concentration of a β-lactoglobulin B protein isoform in bovine milk caused by a synonymous SNP

**DOI:** 10.1186/s12711-022-00711-z

**Published:** 2022-03-16

**Authors:** Stephen R. Davis, Hamish E. Ward, Van Kelly, David Palmer, Alexandra E. Ankersmit-Udy, Thomas J. Lopdell, Sarah D. Berry, Mathew D. Littlejohn, Kathryn Tiplady, Linda F. Adams, Katie Carnie, Alayna Burrett, Natalie Thomas, Russell G. Snell, Richard J. Spelman, Klaus Lehnert

**Affiliations:** 1 Research & Development, Livestock Improvement Corporation, Ruakura Road, Hamilton, New Zealand; 2 ViaLactia Biosciences Ltd., a subsidiary (now closed) of Fonterra Co-Operative Ltd., Fanshawe Street, Auckland, New Zealand; 3 School of Biological Sciences, University of Auckland, Symonds Street, Auckland, New Zealand

## Abstract

**Background:**

Milk samples from 10,641 dairy cattle were screened by a mass spectrometry method for extreme concentrations of the A or B isoforms of the whey protein, β-lactoglobulin (BLG), to identify causative genetic variation driving changes in BLG concentration.

**Results:**

A cohort of cows, from a single sire family, was identified that produced milk containing a low concentration of the BLG B protein isoform. A genome-wide association study (GWAS) of BLG B protein isoform concentration in milk from *AB* heterozygous cows, detected a group of highly significant single nucleotide polymorphisms (SNPs) within or close to the *BLG* gene. Among these was a synonymous *G/A* variation at position + 78 bp in exon 1 of the *BLG* gene (chr11:103256256*G* > *A*). The effect of the *A* allele of this SNP (which we named *B’)* on *BLG* expression was evaluated in a luciferase reporter assay in transfected CHO-K1 and MCF-7 cells. In both cell types, the presence of the *B’* allele in a plasmid containing the bovine *BLG* gene from -922 to + 898 bp (relative to the transcription initiation site) resulted in a 60% relative reduction in mRNA expression, compared to the plasmid containing the wild-type *B* sequence allele. Examination of a mammary RNAseq dataset (n = 391) identified 14 heterozygous carriers of the *B’* allele which were homozygous for the BLG B protein isoform (BB’). The level of expression of the *BLG B’* allele was 41.9 ± 1.0% of that of the wild-type *BLG B* allele. Milk samples from three cows, homozygous for the *A* allele at chr11:103,256,256 (*B’B’*), were analysed (HPLC) and showed BLG concentrations of 1.04, 1.26 and 1.83 g/L relative to a mean of 4.84 g/L in milk from 16 herd contemporaries of mixed (*A* and *B*) *BLG* genotypes. The mechanism by which *B’* downregulates milk BLG concentration remains to be determined.

**Conclusions:**

High-throughput screening and identification of outliers, enabled the discovery of a synonymous *G* > *A* mutation in exon 1 of the *B* allele of the *BLG* gene (*B’*), which reduced the milk concentration of β-lactoglobulin B protein isoform, by more than 50%. Milk from cows carrying the *B’* allele is expected to have improved processing characteristics, particularly for cheese-making.

**Supplementary Information:**

The online version contains supplementary material available at 10.1186/s12711-022-00711-z.

## Background

β-lactoglobulin (BLG) is the major whey protein in bovine milk, representing over 50% of the whey proteins and approximately 10% of the total milk protein pool [1]. BLG is a member of the lipocalin protein family, a large group of diverse proteins that have a hydrophobic, ligand-binding function [2]. BLG is found only in the milk of certain mammalian species, including artiodactyls, and is absent from human milk. The human homolog of BLG is known as the progestagen-associated endometrial protein (encoded by the *PAEP* gene) and has multiple roles in fertility [3]. The functional relevance of BLG in milk remains unknown although its known ligands include retinol, vitamin D, cholesterol and fatty acids [4, 5].

At least 11 protein isoforms of bovine BLG have been described [6] and categorized using an alphabetical nomenclature. The protein isoforms A and B are the most common in dairy cattle breeds and differ by two amino acids (Asp64/Val118 and Gly64/Ala118 in A and B, respectively) in the mature secreted protein. Furthermore, in a study of genomic DNA from 22 proven bulls in Holland, 50 DNA polymorphisms were identified in the coding and promoter regions of the *BLG* gene. More than 40 of these polymorphisms were in complete linkage disequilibrium (LD) with the SNP defining the A and B protein isoforms [7]. Bedere and Bovenhuis [8] confirmed that the *A* and *B* alleles explain most of the variation in BLG concentration in bovine milk. The lower concentration of the BLG B protein isoform in milk compared to the A protein isoform was shown to be associated with a reduced expression of the *BLG B* allele of ~ 60–90% compared to that of the *A* allele [9, 10].

Since differences in milk composition are associated with *BLG* genetic variants, selection for specific polymorphisms to improve milk functionality and processing characteristics can be considered. Variation in the concentration of BLG in milk is inversely associated with variation in casein number, which is calculated as a proportion of total protein content. Thus, the low-concentration BLG B protein isoform is associated with a greater casein number [10] which, in turn, is associated with enhanced cheese yield [11, 12]. In addition, milk with a lower BLG content may have advantages in terms of cheese processing since the more highly expressed *BLG A* allele has been implicated in increased plant-fouling during the manufacturing of ultra-high temperature (UHT) milk compared to the *BLG B* allele [13].

Schopen et al. [14] reported that SNPs on *Bos taurus* (BTA) chromosomes 6, 11 and 24 were associated with BLG concentration in milk, and Gambra et al. [15] reported that SNPs in nine genes, including genes on BTA6 and 11 (but not BTA24) were associated with BLG concentration. In Swiss Brown cattle, Braunschweig and Leeb [16] described a novel SNP that is associated with a low-concentration variant of BLG and is due to a unique *C* to *A* transversion/substitution, which is located 215 bp upstream from the translation start site of the *BLG* gene, although causation was not proven.

The objective of the current study was to screen a population of dairy cattle for milk with extreme (high or low) concentrations of BLG and to identify genetic variant(s) that are responsible for these observed extreme variations. This screen identified a population of cows in the New Zealand (NZ) herd that carried a sub-variant of the *B* allele, which results in an unusually low concentration of BLG in milk. Furthermore, we describe the genetic basis of this low BLG concentration and highlight a candidate causative mutation that underlies this effect.

## Methods

The experimental steps undertaken in this study were as follows:

1. Screening of milk samples from commercial dairy herds for concentrations of A and B isoforms of BLG using high-throughput, liquid chromatography/mass spectrometry.
2. Identification of a population of cows producing milk with a low concentration of BLG B protein isoform and linked to the same sire family.
3. Demonstration of segregation of the low BLG B protein isoform in milk from daughters of a putative sire-carrier, homozygous for the *B* allele of *BLG.*
4. Identification of putative causal variants of the low BLG B isoform from a GWAS of total BLG, followed by GWAS of the concentrations of the BLG B isoform in milk from heterozygous *AB BLG* cows.
5. Determination from in vitro gene cloning experiments that a putative causal SNP decreased the expression of a *BLG*/*luciferase* reporter construct encompassing part of the promoter sequence, exon 1, intron 1 and part of exon 2 of *BLG*.
6. Demonstration of the association of *BLG* mRNA expression with the causal SNP by RNAseq.
7. Evaluation of the impact of the causal SNP on milk composition.

In the following text, the variant chr11:103256256*G* > *A* in exon 1 of the *B* allele of *BLG* is referred to as *B’*.

### Milk sampling

In total, 10,641 milk samples, from individual cows, were collected from 40 commercial dairy herds (Friesian, Jersey and their crossbreds). All milk samples were collected from October to December, corresponding to mid-lactation (approximately 70 to 130 d post-calving) for spring-calving dairy herds in New Zealand. Milk samples from all cows were collected using standard herd-testing procedures in New Zealand (LIC, Newstead, Hamilton, New Zealand). Sub-samples (1 mL) of the aqueous phase were taken from a composite am/pm milk sample, after overnight storage at 4 °C, and stored in 96-well plates at − 80 °C, until analysis. Farms were selected primarily because they were suppliers to Fonterra Co-operative Group Ltd., (Fanshawe St., Central Auckland 1010, New Zealand) and users of LIC genetics. Cow-level information, including breed, pedigree, age, calving date and milk composition was retrieved from herd records held in the LIC herd management database (MINDA®, LIC, Hamilton, New Zealand).

### Screening of the milk samples for the BLG A and B protein isoforms

For the initial screening of the 10,641 milk samples, a mass spectrometry method was used to quantify the concentrations of the BLG A and B protein isoforms in milk. First, casein was removed by adding acetic acid to skim milk samples to a final concentration of 1%, followed by centrifugation for 5 min at 1000* g*. The resulting whey fraction was diluted 800-fold with a 30% methanol/0.1% acetic acid/69.9% water solution, prior to analysis for BLG by mass spectrometry. Diluted whey samples were analysed by flow-injection analysis/mass spectrometry using an Agilent Technologies (Palo Alto, CA, USA) 1100 series liquid chromatograph coupled to an Agilent Technologies 6310 ion-trap mass spectrometer with an electrospray ionisation interface. Samples (30 µL) were injected into a stream of 30% methanol/0.1% acetic acid/69.9% water and were delivered to the mass spectrometer at 100 µL/min. The mass spectrometer was operated in positive ion mode and mass spectra were collected between 1400 and 1900 m/z. The level of the BLG A isoform was calculated from the signals at 1413.4, 1531.1, 1670.2 and 1837.1 m/z corresponding to [M + 13H]^13+^, [M + 12H]^12+^, [M + 11H]^11+^ and [M + 10H]^10+^, respectively, and the level of the BLG B isoform was calculated by summing the signals at 1406.8, 1523.9, 1662.4 and 1828.5 m/z corresponding to [M + 13H]^13+^, [M + 12H]^12+^, [M + 11H]^11+^ and [M + 10H]^10+^, respectively. The signals used for quantification of the BLG B protein isoform did not discriminate the B protein from the C protein isoform of BLG. However, the frequency of the *C* allele is low (< 1.7%) in the NZ dairy cow population (estimated from a subset of 166,000 genotyped cows imputed to sequence). Ultimately, the cows carrying the *C* allele were excluded based on genotyping data (see below), as a relevant source of variation.

The specific signals for the BLG A and B isoforms were characterized using BLG standards (Sigma-Aldrich, St. Louis, MO, USA) and the concentrations were expressed as arbitrary units (arb. units). For guidance, it was estimated that 80 arb. units (the average concentration found in milk from homozygous *BB* cows) were equivalent to 3.4 g/L of BLG, which is the mean concentration of the BLG B isoform measured in NZ milk samples from homozygous *BB* cows [17]. Similarly, the mean concentration of the BLG A isoform in milk samples from homozygous *AA* cows in NZ was 4.8 g/L, which is equivalent to an average concentration of 180 arb. units.

### Validation of BLG mass spectrometry screening assay

The concentration of BLG in milk from 40 cows in mid-lactation was also determined by high-performance liquid chromatography (HPLC) as described by [18] and was found to be highly correlated with the BLG concentration determined by the LC/MS screen (see Additional file 1: Fig. S1).

### Blood and tissue sampling for genotyping

Samples of ear tissue (by punch), blood or semen, depending on ease of sampling for each situation specified below, were used for DNA extraction and genotyping. When required, blood samples were collected from the coccygeal vein by venipuncture into EDTA-coated vacutainer tubes. Whole blood was stored at − 20 °C until analysis. DNA was extracted from blood and semen samples using a standard phenol–chloroform method [19] and from ear tissue samples using the Qiagen BioSprint 96 DNA extraction kit (Qiagen, Valencia, CA, USA), following the manufacturer’s instructions.

### Genome-wide association studies of BLG phenotypes

Genome-wide genotypes were available for 4140 of the 10,641 screened cows with BLG phenotypes (BLG protein isoform concentrations) and 2091 of these were heterozygous (*AB*) at the *BLG* locus. Genotypes had previously been generated by Neogen (Lincoln, NE) on the Illumina Bovine SNP50 panel. The low-density SNP-chip genotypes were imputed to sequence using a dataset of 16.1 million variants identified from whole-genome sequences of a reference population consisting of 306 Holstein–Friesian, 219 Jersey and 717 crossbred cattle in the NZ dairy herd (males and females). The sequence imputation pipeline is described in [20–22]. BLG phenotypes (BLG protein isoform concentrations) from mass spectrometry were adjusted using a mixed linear model in the R software [23] with fixed effects included for birth year and breed, and random effects included for herd and plate number.

A GWAS was performed for total BLG concentration in the milk from 4140 cows in the BLG LC/MS screen for which genotypes were available. Furthermore, GWAS for each BLG A and B isoform concentrations (arb. units) in the milk from 1966 heterozygous *AB* cows were also performed by conducting a mixed linear model analysis using the GCTA v. 1.93.0 beta software package [24] across 16,122,289 imputed sequence variants. Population structure was accounted for by using a genomic relationship matrix (GRM) derived from 45,135 SNPs, constructed from 50 k-resolution genotypes, as described in [20–22]. A leave-one-chromosome-out (LOCO) approach was used, in which separate GRM were derived for each chromosome based on SNPs from the other chromosomes only. Significance levels were evaluated using a Bonferroni correction, with all the tests across 16,122,289 variants and three phenotypes being considered as independent traits. Based on a genome-wide threshold of α = 0.01, this resulted in a nominal *p*-value of 2.07e−10.

All genomic positions presented in this paper are based on the ARS-UCD1.2 reference assembly (https://www.ncbi.nlm.nih.gov/assembly/GCF_002263795.1).

### Segregation of the BLG *B* alleles in the daughters of Sire 99

To investigate the segregation of the *BLG B* alleles, a new set of 576 milk samples was collected from the daughters of a single bull, known as Sire 99, that was identified in this study as a carrier of both the *B* and *B’* alleles of the *BLG* gene. These cows were sampled during mid-lactation from approximately 220 commercial farms. The distribution of the concentration of the BLG B protein isoform in the milk samples of a subset of *AB* heterozygous daughters of Sire 99 (n = 234) was determined from mass spectrometry analysis. All genotypes were inferred from the mass spectrometry analysis and later confirmed by PCR (see below).

### In vitro* BLG*-promoter assay

Using a luciferase reporter assay, the impact of the chr11:103256256G > A SNP (*B’*) on *BLG* expression was studied in vitro in two cell lines, the human mammary adenocarcinoma (MCF-7**)** and the Chinese Hamster Ovary (CHO-K1**)** that were obtained from the American Type Culture Collection, ATTC, Manassas, VA, USA. The 1819 bp genomic regions comprising 922 bp of the *BLG* promoter (relative to the transcription initiation site of transcript NM_173929.3), exon 1, intron 1, and the 5′ 139 bp of exon 2, were PCR-amplified from genomic DNA prepared from *BB* and *B’B’* homozygous animals using the Expand Long Range dNTPack (Roche Diagnostics NZ Ltd, Auckland, New Zealand), with the forward primer 1 (5’- **CTCGAG**AGATCTCCACAGCCCGCTGGGTACTGATGCCC) and reverse primer 2 (5’- **AAGCTT**CAGCTGATTTCTGCAGCAGGATCTCCAGGTCGCC to amplify the *B*-allele) or reverse primer 3 (5’- **AAGCTT**GTCGACATTTCTGCAGCAGGATCTCCAGGTCGCC to amplify the *B’*-allele), respectively. All primers were designed using the Primer-BLAST web tool (https://www.ncbi.nlm.nih.gov/tools/primer-blast/). To facilitate subcloning and diagnostic restriction analysis, recognition motifs for the XhoI and BglII restriction enzymes were added to the 5’-end of the forward primer 1, those for PvuII and HindIII to the reverse primer 2, and those for SalI and HindIII to the reverse primer 3, respectively (indicated in bold and underlined, respectively, in the above primer sequences). PCR products were cloned into pCR2.1-TOPO (Life Technologies New Zealand Ltd., Auckland, New Zealand). *BLG* allelic sequences were verified by Sanger sequencing, followed by subcloning of the XhoI/HindIII fragments into the promoter-less luciferase pGL4.16 vector (Promega Corp., Madison, WI) using the Rapid DNA Ligation kit (Roche) to create plasmids B and B’, which drove luciferase expression under the control of the proximal promoter and 5’ regions of the *BLG B* and *B’* alleles, respectively.

MCF-7 and CHO-K1 cells were grown in MEM-alpha medium, supplemented with 10% fetal bovine serum, 1 mM sodium pyruvate, 1 × penicillin/streptomycin (all Life Technologies). For the MCF-7 cells, the cell culture medium was supplemented with 10 μg/mL insulin (Sigma-Aldrich). Cells from both cell lines were grown to 80% confluence before transfection with the FuGene Transfection Reagent (Roche). Transfections were carried out using 6 µg plasmid DNA per 2 µL of the FuGene reagent. Transfection efficiency was normalised between wells by inclusion of the Renilla luciferase pGL4.73 vector (Promega) at a 9:1 ratio. Prior to harvest, cells were grown for a further 48 h in MEM-alpha medium, supplemented with 10% fetal bovine serum, 1 mM sodium pyruvate, 1 × penicillin/streptomycin (all from Life Technologies), 10 μg/mL insulin, 5 μg/mL prolactin and 1 μg/mL hydrocortisone (all from Sigma-Aldrich). Cells were harvested by removing the medium and rinsed with phosphate-buffered saline (PBS) solution, and then lysed with Passive Lysis buffer (Promega).

The luciferase assay was performed using a Dual Luciferase Reporter Assay (Promega). Luminescence was measured using the EnVision Multilabel Plate Reader (PerkinElmer, Boston, MA). Cellular lysate (20 μL) was dispensed into a 96 well plate, 100 μL of LAR II (Promega) was added to each well and the luminescence measured five times. Subsequently, 100 μL of Stop & Glo Reagent (Promega) was added to each well and the luminescence was again measured five times. Measurements of cellular luciferase activity were averaged per well and normalised by the corresponding Renilla luciferase measurement giving a ratio of Luciferase: Renilla luciferase. Results, from 12 independent transfections for each plasmid, were averaged.

### In vivo* BLG* expression

Tissue was sampled from the lactating mammary glands of 391 cows with known *BLG* genotypes by needle biopsy, as previously described [25], and total RNA was extracted. The animals were 3- and 4-year-old Holstein–Friesian $\times$ Jersey cows (FJX) in mid-lactation. cDNA libraries were produced using the TruSeq RNA Sample Prep Kit v2 (Illumina) and were sequenced by the Australian Genome Research Facility (AGRF; Melbourne, Australia) on an Illumina HiSeq 2000 sequencer (see [26, 27] for details). To reduce mapping bias in downstream allele-specific expression (ASE) analyses, sequence reads were mapped to a masked version of the ARS-UCD1.2 *Bos taurus* genome, where known variants were replaced by bases that differed from both the reference and known alternative alleles. Mapping was performed using STAR (version 2.7.0) [28], following the multi-sample two-pass approach described in the STAR manual.

### Genotyping

SNPs were called from the mapped RNA-seq reads using HaplotypeCaller from the GATK software package (version 4.1.8) [29, 30] following the application of the GATK tools markDuplicates and SplitNCigarReads [31]. Genotypes of each animal for the three *BLG* SNPs p.Glu64Asp (rs110066229), p.Val118Ala (rs109625649), and p.Leu26Leu (rs209645844) were used to determine the *BLG* genotype classes shown in Fig. 4.

### RNA abundance measurement

To evaluate the effects of each genotype on *BLG* expression, total expression was determined using the Stringtie software [32] with the mapped RNA-seq reads and normalized for each animal through adjustment for total read count of that animal relative to the average read count for all animals. Subsequently, an ASE methodology was used to further examine the effect of each genotype on *BLG* expression. The “count” method from IGVtools [33] was used to determine the fractions of RNA-seq reads that contain each allele at positions Chr11:103,256,256 (rs209645844; to distinguish the *B* and *B’* alleles) and Chr11:103,259,232 (rs109625649) to distinguish the *A* and *B* alleles. Reads from secondary alignments were excluded. The expression of the *B’* allele relative to the *A* and *B* alleles was calculated as the ratio of reads containing the *B’* allele in *AB’* (n = 19) and *BB’* (n = 14) genotyped animals relative to the equivalent B protein carrier classes (AB and BB).

### Verification of the *BLG *chr11:103256256*G* > *A* SNP effect and its impact on milk composition

Approximately 700 cows that were daughters of *B’* carrier sires and whose dams were also sired by *B’* carrier sires were identified on commercial farms. Blood samples were collected from 510 of these cows across 350 commercial herds, and the DNA extracted for genotyping. The *B’* SNP (rs209645844) and the SNPs defining the A, B (rs11066229 and rs109625649), and C (rs210096472) protein isoforms, were genotyped by the Australian Genome Research Facility (AGRF; Queensland, Australia). The AGRF custom SNP genotyping service used the single base extend method (SBE—Iplex GOLD chemistry), analysed on the Sequenom Compact Mass spectrometer.

Composite am/pm milk samples from the 510 genotyped cows were analysed by mid-infrared spectroscopy (FT120, Foss, Hillerod, Denmark) and their casein content was determined by acid precipitation [34]. Samples were taken at peak lactation, and those from cows with a somatic cell count greater than 400,000 in their milk and those without genotypes were excluded. BLG concentration was determined by mass spectrometry, as described above. Milk composition data from *AA*, *BB* and *B’B’ BLG* genotypes were analysed by ANOVA in JMP Version 8.0. (SAS Institute. Cary, NC, USA).

## Results

### Screening of BLG A and B isoform concentrations in milk samples

**Fig. 1:**
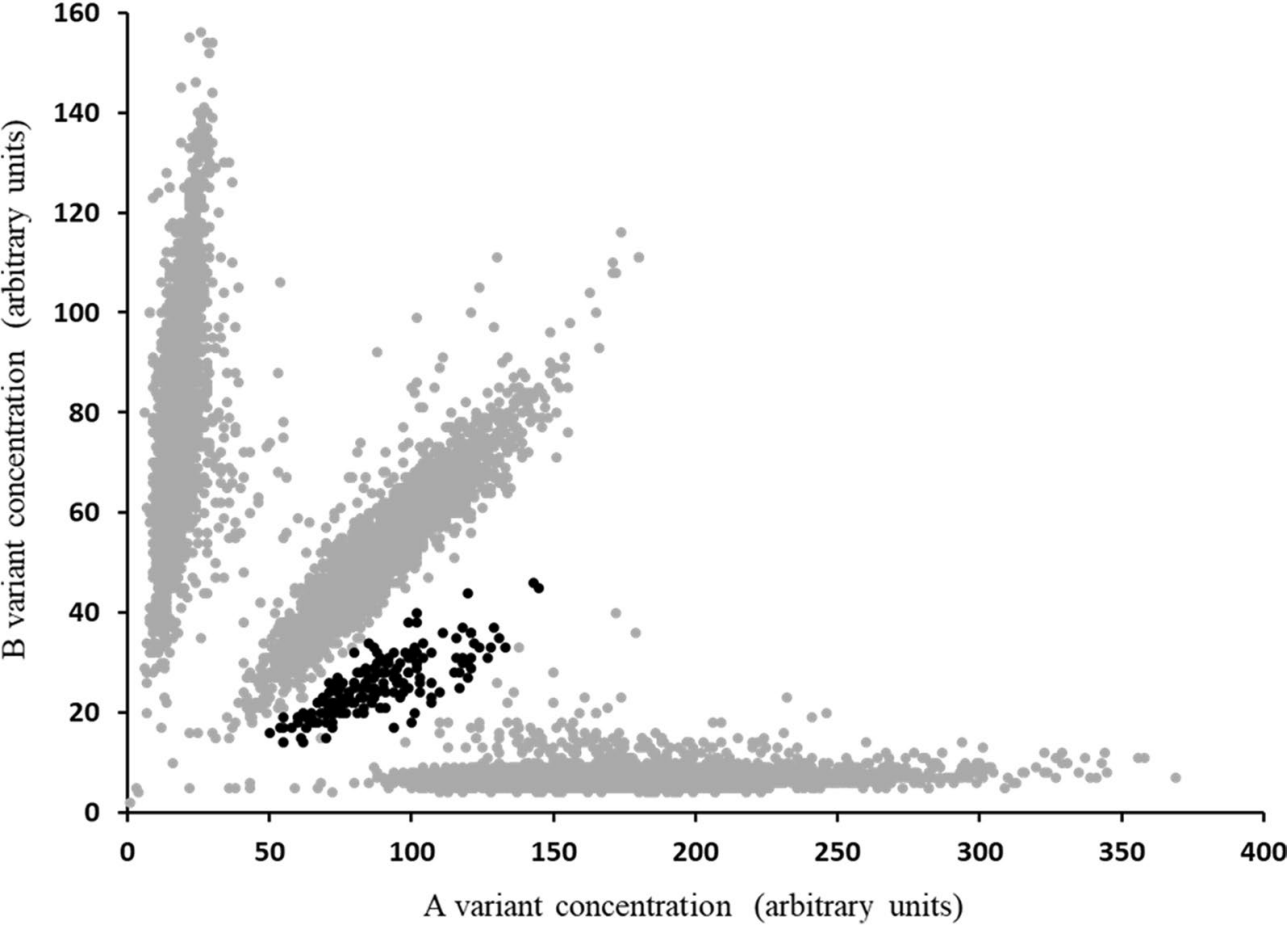
Identification of BLG A and B protein isoform populations, in milk samples collected from 10,641 cows during mid lactation. By plotting the B vs. A protein isoform concentrations of BLG (determined by mass spectrometry) milks containing AA, BB and AB protein isoforms can be distinguished. Black data points highlight the milk samples containing both protein isoforms but with a markedly lower concentration of the BLG B isoform

The LC/MS screen of milk samples from 10,641 individual cows enabled the quantification of BLG A or B protein isoforms. A plot of the concentration of the BLG B vs. A protein isoforms in the screened milk samples is in Fig. 1 and a cohort of almost 200 milk samples containing a relatively low concentration of the BLG B protein isoform was identified.

The cohort of cows that expressed the BLG B isoform at a low concentration was selected for further investigation. Heterozygous *AB BLG* cows (n = 180; Fig. 1) carrying the low-concentration *B BLG* allele were selected and sire frequency in this group was evaluated. These 180 cows were sired by 126 different sires, however, the five most frequently observed sires with a total of 25 daughters, were the sons or grandsons of the same sire. Among another group of 180 *BB* homozygous cows with the lowest milk BLG concentration, the five most frequently represented sires (out of 113 in the selection)) collectively had 42 daughters and all shared the same common ancestral sire as the *AB* heterozygous population of cows with a low concentration of BLG in milk. The top three sires were common to both the *AB* and *BB* groups.

One sire, which historically, was frequently used in New Zealand, (hereafter referred to as Sire 99), had 18 daughters in the low-concentration BLG B isoform *BB* group and seven daughters in the low-concentration BLG B isoform in the *AB* group and was a grandson of the common ancestor. Daughters of this sire were identified for further study with the objective of deciphering the mechanism responsible for the low-concentration BLG B isoform. Sire 99 was previously shown by genotyping to be homozygous for the *B* allele of *BLG* and was later confirmed to be heterozygous for the *B’* allele. Hence, *AB* heterozygous daughters of Sire 99 could be used to investigate segregation of BLG B isoform concentration in milk.

**Fig. 2:**
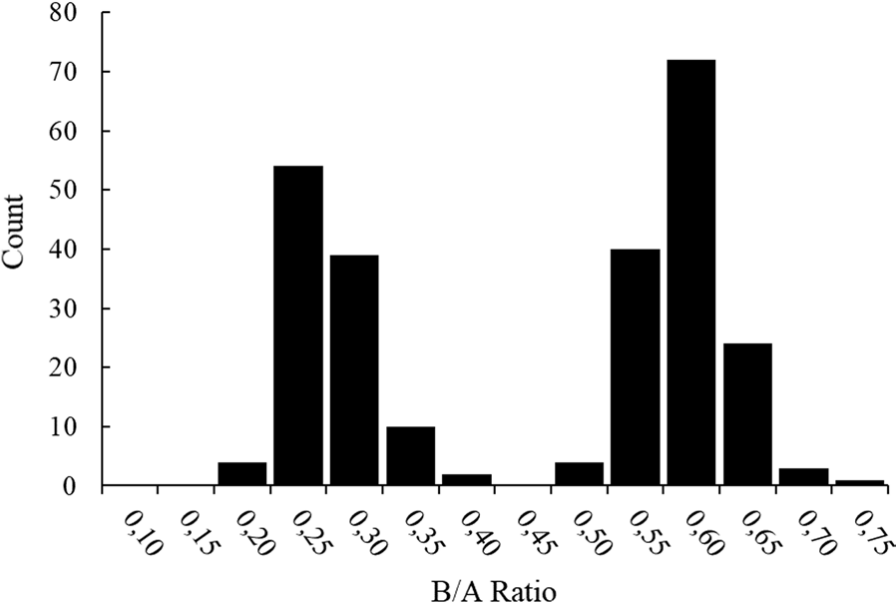
Distribution of BLG B isoform concentrations in milk samples from *AB* heterozygous daughters (n = 234) of Sire 99. Results are expressed as the B/A ratio based on the quantification of BLG by mass spectrometry. Segregation of the B isoform populations is shown

Milk samples were collected from 576 daughters of Sire 99 in commercial herds and analysed by LC/MS to determine the concentrations of BLG A and B protein isoforms. The ratios of BLG B:A concentrations in milk from heterozygous *AB* daughters (n = 234) are shown in Fig. 2. Expressing the results as a B:A ratio allowed determination of the segregation of the BLG B isoform concentrations and revealed two distinct populations (Fig. 2), which indicated that this sire was heterozygous for an allele (or alleles) that regulates the BLG B protein isoform concentration. The mean concentration of the BLG B isoform in the segregating groups (n = 133 *AB* and n = 101 *AB’*) was equal to 54.0 ± 8.7 (SD) and 25.9 ± 6.1 arb. units, respectively.

### Genome-wide association studies with total, and A and B isoform concentrations of milk BLG

In the GWAS of total BLG concentration, 22,888 SNPs were significantly associated, which were all located on BTA11 in the region ranging from 82.4 to 106.9 Mb. Two SNPs which defined the two amino acid polymorphisms associated with the A or B protein isoforms ranked 83 and 102 (based on –log_10_(*p*-values)) among all significant SNPs (see Additional file 2: Table S1). Among the genotyped animals, the *AB* heterozygotes (n = 2091) were selected, and the GWAS was repeated using the concentration of either the BLG A or B protein isoforms, determined by mass spectrometry. In total, 1450 SNPs were significantly associated with BLG B isoform concentrations, of which 1449 were on BTA11 (the top 250 SNPs are shown in Additional file 2: Table S2). The 1330 SNPs that were most significantly associated with BLG B isoform concentration were located on BTA11 in the region between 87.7 and 107.0 Mb, with –log_10_(*p*-values) ranging from 1.91e−58 to 4.47e−11. Among these, a synonymous SNP, p.Leu26Leu (rs209645844) was located at position + 78 relative to the start site of the *BLG* gene. This SNP had a minor allele frequency of 1.87% in the dataset of *AB* heterozygous cows and ranked 2472 for significance (−log_10_(*p*-values)) among all SNPs in the GWAS across all animals for total BLG. Most importantly, Sire 99 was heterozygous for this SNP. Five other SNPs had a similar level of significance and were all located downstream of this synonymous SNP. Two of these were in introns and three were outside the UTR region of the *BLG* gene (see Additional file 2: Table S2). Although one significant association with BLG B isoform concentration was observed on chromosome BTA3 (position 67,051,732), evidence for any additional QTL was weak, due to a lack of support from adjacent SNPs that were in LD. One hundred and forty-one SNPs were significantly associated with variation in BLG A isoform concentration and were all located on BTA11.

### Functional consequence of the *BLG B’* allele in vitro

**Fig. 3:**
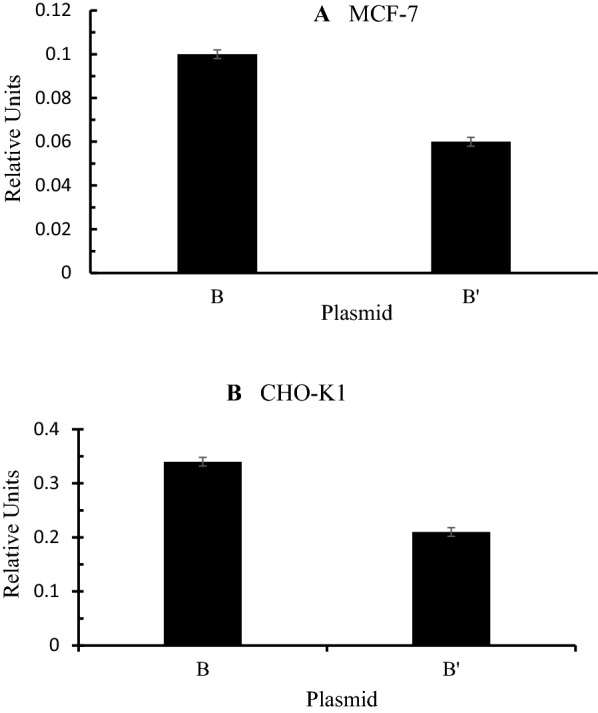
In vitro gene expression of a *BLG*-luciferase reporter construct in MCF-7 **a** and CHO-K1 **b** cells. Constructs with the wild type G allele of rs209645844 (B allele) and an analogous construct containing the chr11: 103256256G > AA allele (B’ allele) are shown. The genomic regions used in the reporter constructs included 881 bp of the *BLG* promoter (relative to the transcription initiation site of transcript NM_173929.3), exon 1, intron 1, and 139 bp of exon 2. Results are means from 12 separate transfections undertaken for each construct. Bars are standard errors of the mean

Given that, among the top candidate genetic variants for a low-concentration BLG, a synonymous mutation (p.Leu26Leu) was found, it was of interest to investigate whether this mutation might mediate an impact on *BLG* expression through modification of mRNA stability, splicing, or some other molecular mechanism. To test this hypothesis, we undertook reporter assays in two mammalian cell lines. Luciferase reporter activity was reduced to about 60% in cells containing the luciferase plasmid under control of the promotor and 5’ region of the *BLG B’* allele, compared to the plasmid expressing luciferase under the control of the analogous region from the *BLG B* allele in both MCF-7 and CHO-K1 cell lines (Fig. 3).

### In vivo expression of *BLG*

**Fig. 4:**
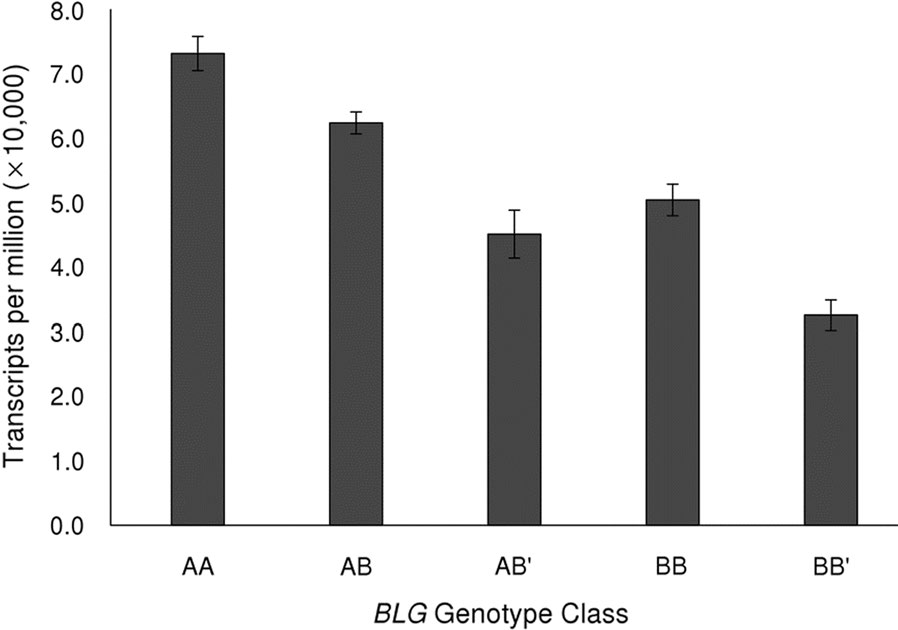
Gene expression of *BLG* in mammary tissue obtained from biopsies of cows (n = 392) in mid-lactation. Normalized read counts from RNA-Seq of mammary biopsies are shown for each *BLG* genotype class (n = 98, 191, 19, 70 and 14 for *AA*, *AB*, *AB’*, *BB* and *BB’* classes, respectively). There were no *B’B’* homozygous individuals in the dataset

The expression of *BLG* mRNA in mammary biopsies from cows of different *BLG* genotypes is shown in Fig. 4. The majority of the RNA-seq animals were genotyped as *AB*, and no *B’B’* animals were detected in the sampled population. *BLG* mRNA levels observed in the *AA* animals were 1.45 (SD = 0.044 by bootstrapping) times greater than those in the *BB* animals, and the *BLG* mRNA levels in the *BB* animals were 1.55 (SD = 0.071) times greater than those in the *BB’* animals.

The read counts of each genotype of the heterozygous animals were used to determine allele-specific effects of each allele on *BLG* mRNA levels. The mean expression of the *B* allele was 64.2 ± 1.2% of that of the *A* allele when comparing milk samples from homozygous *AA* and *BB* cows, and the mean of expression of the *B’* allele was 38.2% of that of the *B* allele when comparing milk samples from 19 heterozygous *AB’* and 190 heterozygous *AB* cows. Expression of the *B’* allele was equal to 41.9 ± 1.0% of that of the *B* allele in 14 heterozygous *BB’* cows.

### Effects of *BLG* genotype on milk composition

Among the 510 cows used for genotyping and assessing milk composition, 28 were homozygous for the *B’*, 64 homozygous for the *B*, and 29 homozygous for the *A* allele of *BLG*. Milk samples from seven cows that carried the *C* allele of *BLG* were excluded. Milk composition was very similar between the groups with a small but significant (*P* < 0.05) increase in casein number (casein calculated as a percentage of true protein; Table 1) in milk from the homozygous *B’* cows. Differences between the major milk components (fat, crude protein, true protein, and lactose) by genotype, were not statistically significant.

**Table 1:** Milk composition by *BLG* genotype group

Milk component	*BLG* genotype group		
	*B'B'* (n = 28)	*BB* (n = 64)	*AA* (n = 29)
Fat (g/L)	41.0 (4.0)	44.6 (3.1)	43.6 (4.2)
Crude protein (g/L)	34.9 (0.7)	34.9 (0.4)	35.2 (0.6)
True protein (g/L)	32.3 (0.7)	32.3 (0.4)	32.8 (0.6)
Lactose (g/L)	47.7 (0.4)	47.1 (0.4)	47.2 (0.6)
Casein (g/L)	27.4 (0.6)	27.0 (0.3)	27.2 (0.5)
Casein (% true protein)	0.85 (0.004)^a^	0.84 (0.003)^b^	0.83 (0.004)^c^
β-lactoglobulin concentration. arb. units	24 (1.0)^a^	46 (1)^b^	83 (4)^c^

Composite (am/pm) milk samples were collected from Friesian/Jersey crossbred cows of defined *BLG* genotype, in mid-lactation and major components analysed by mid infra-red spectroscopy. *BLG* was quantified by mass spectrometry and casein by wet chemistry. Figures in parentheses are standard errors of the mean (SEM). Significant differences (*P* < 0.05) among genotype groups, by analysis of variance, are indicated by differing letters in superscripts

Absolute concentrations of BLG were also determined by HPLC in milk samples collected at mid-lactation from three cows that were homozygous for the *B’* allele. The mean BLG concentration was 1.38 ± 0.24 (SEM) g/L. In a group of 16 contemporary cows in the same commercial herd, matched for age, the mean BLG concentration in milk was 4.84 ± 0.29 g/L.

## Discussion

Our results demonstrate the utility of a high-throughput screening method for milk BLG, which led to the discovery and characterization of a relatively rare, genetic variant with a significant impact on milk protein composition. A synonymous SNP, in exon 1 of the *BLG* gene, was identified by this screening method and was determined to be responsible for a low concentration of the BLG B protein isoform in milk. We refer to this *BLG* genetic variant that includes the *A* allele of this SNP, as *B’*. This variant is linked to a sire family that has been widely used in the New Zealand dairy industry. Evidence that the *A* allele at this SNP is the causative variant includes the association of the *B’* variant with a low concentration of the BLG B protein in milk, reduced expression of a *BLG*-luciferase reporter in a cell culture system, and reduced *BLG* mRNA levels in lactating mammary tissue. Furthermore, this SNP is among the five most significant SNPs identified in a GWAS of BLG B protein concentration in milk.

Among the possible mechanisms by which the *B’* allele might reduce *BLG* gene expression are the disruption of an exonic splice enhancer, the modification of mRNA stability or the disruption of a regulatory promoter element overlapping with *BLG* exon 1. Previous work by [35] described positive and negative, intragenic, regulatory elements in the ovine *BLG* gene in transient transfection experiments in cell lines and in transgenic mice. In particular, removal of sequences within exon 1, intron 1 and exon 2 of *BLG* abolished *BLG*–directed expression of human serum albumin in vitro, although not in vivo. Promoter elements are typically found immediately upstream of the transcription start site, but they have also been observed within the first exon of at least one gene [36, 37]. We examined the *BLG* exon 1 for the presence of transcription factor binding sites, using the R packages JASPAR2018 [38] and TFBSTools [39], but we did not identify any binding sites that were affected by the *B’* allele, thus there is no evidence that this allele reduces *BLG* expression by compromising promoter elements within the first exon.

Another mechanism that might explain the effect of the *B’* allele is a reduction of the rate of intron excision, which thus decreases the rate of production of mature *BLG* mRNA. To investigate this, we analysed the 3′ 40 bp of exon 1, using the software package RESCUE-ESE [40, 41] and both the human and mouse exonic splice enhancer (ESE) hexamer sets. No effect was observed for the *B’* allele when using the human hexamers. However, it is interesting to note that the *B’* allele removed three ESE elements that were detected using the mouse hexamer set (seven other elements were common to the *B* and *B’* alleles). Nevertheless, no aberrant splicing events were detected when we examined the RNA-seq alignments generated from animals carrying the *B’* allele, so it is unlikely that this synonymous SNP acts via a splicing-mediated mechanism.

Several studies have focussed on the association between SNPs and milk BLG concentration, based on the observation that the BLG A isoform was associated with ~ 50% more BLG protein in milk than the B isoform [10, 42]. At least two different haplotypes within the *A* and *B BLG* alleles were associated with variation in BLG concentration suggesting that the *BLG* chromosomal region contains further mutations affecting both A and B protein isoform concentrations [8]. Furthermore, ten polymorphic sites in the 3′ 733 bp of the promoter region of *BLG* were identified that were specific either to the *A* or the *B BLG* alleles and associated with differential expression of *BLG* [43]. In another study, polymorphisms located within an AP2 binding site of the promoter region of *BLG* were implicated in allele-specific differences in *BLG* gene expression [44].

Braunschweig and Leeb [16] identified a novel SNP associated with a low-concentration variant of BLG in milk from Swiss Brown cattle, which corresponded to a unique, *C* to *A* transversion, 215 bp upstream from the translation start site, although causation was not proven. This variant has not been observed in the genotyped or sequenced New Zealand dairy population.

The *B’* allele of the *BLG* gene has already been reported in a comparison of *BLG* sequences from 19 cow breeds [45]. It was present at a frequency of 2–3.5% in Angus, Dexter, SDM (black and white Danish dairy) and Jersey cattle and of 11% in Hereford cattle. The best estimates of *B’* allele frequency in New Zealand dairy cattle are based on a genotyped population of 160,000 animals and indicate a 1–2% allele frequency in Friesian and Friesian X Jersey breeds. Direct genotyping of the chr11:103256256*G* > *A* polymorphism in DNA extracted from semen stocks for 1378 highly-used sires in New Zealand, identified 49 sires that carry the *B’* allele. Based on pedigree data, all these sires could be linked back to a foundation sire in New Zealand that began commercial use in the 1990’s.

The *B* allele of *BLG* is associated with a lower BLG concentration [10, 46], a greater casein number [10, 44], a somewhat longer renneting time and lower heat stability [47] of milk. Boland and Hill [11] showed that the preferential selection of animals carrying the *B* allele of *BLG*, resulted in increased milk casein and cheese yield per kg of milk protein compared to animals carrying the *A* allele. Thus, selection for the *BLG B* allele will result in a greater casein number and increased cheese yield, and selection for the *BLG B’* allele is expected to improve casein number and cheese yield still further, compared to milks containing the A and B protein isoforms.

The BLG B protein isoform has several other advantages in terms of milk processing characteristics which the *B’* allele may improve further. Under the effect of heat, a reactive sulphydryl group within the BLG protein is exposed, which enables cross-linking of BLG with other proteins, particularly κ-casein. A reduction in BLG concentration in milk may facilitate a further reduction in plant fouling rates [13].

BLG is a major allergen in milk [48, 49] but it is unlikely that the allergenicity of milk produced by cows carrying the *B’* allele would be altered by the resulting reduction in BLG concentration. To improve the allergenic properties of cows’ milk, it would be necessary to eliminate BLG from milk through gene editing [50] or to detect a “natural null” allele using similar screening methods. However, as BLG is critical for gel formation in some milk products [51], reducing the expression of *BLG* may have some potentially negative consequences for milk processing, which will require more detailed investigation.

## Conclusions

A targeted, high-throughput screen of milk samples from cows in commercial herds has enabled the identification of a synonymous SNP, in exon 1 of the *BLG* gene, encoding the BLG B protein isoform. This SNP was associated with a substantial reduction in the expression of the *BLG* gene and an approximately 70% lower BLG concentration in the milk of homozygous carriers. Milk from cows that carry the *B’ BLG* allele have a greater casein number and may have improved processing characteristics. This SNP has not been detected in the *BLG* allele coding for the A protein isoform. The causal SNP was identified in a sire family that has been used in dairy cattle breeding in New Zealand for the past 30 years. Currently, the estimated frequency of this *B’ BLG* allele in the Friesian and Friesian $\times$ Jersey dairy population is 1–2%. The precise mechanism through which this SNP down-regulates *BLG* expression and decreases BLG secretion into milk remains to be determined.

## Supplementary Information

**Additional file 1:** **Figure S1.** Correlation between the concentrations of BLG A and B protein isoforms obtained by the mass spectrometry (MS) screening method with those obtained by a high-performance liquid chromatography (HPLC) method [18]. Cows (n = 40) were a mix of heterozygous *AB* and homozygous *AA* and *BB* individuals.

**Additional file 2:** **Table S1**. GWAS results for the top 250 SNPs in the region of the *BLG* gene that were associated with total BLG concentration in milk (n = 4140). Two SNPs that differentiate the A and B protein isoforms are highlighted. **Table S2.** GWAS results for the top 250 SNPs in the region of the *BLG* gene that were associated with BLG B isoform concentrations in milks from heterozygous *AB* animals (n = 1966). The top SNP (highlighted) is B’ (chr11: 103256256G > A).

## Data Availability

GWAS data have been uploaded, in part, as additional tables. Further genome-wide data and ancillary information are available upon reasonable request following execution of a data transfer agreement, and with permission of Livestock Improvement Corporation.
