# Supplementary material for: Screening for phenotypic outliers identifies an unusually low concentration of a β-lactoglobulin B protein isoform in bovine milk caused by a synonymous SNP
Source: Genet Sel Evol. 2022 Mar 16;54:22. doi: 10.1186/s12711-022-00711-z (PMC8925192; doi:10.1186/s12711-022-00711-z)
Supplement: Supplementary file 1 — Additional file 1: Figure S1. Correlation between the concentrations of BLG A and B protein isoforms obtained by the mass spectrometry (MS) screening method with those obtained by a high-performance liquid chromatography (HPLC) method [18]. Cows (n = 40) were a mix of heterozygous AB and homozygous AA and BB individuals. [file 12711_2022_711_MOESM1_ESM.docx]

**Additional File S1.**
